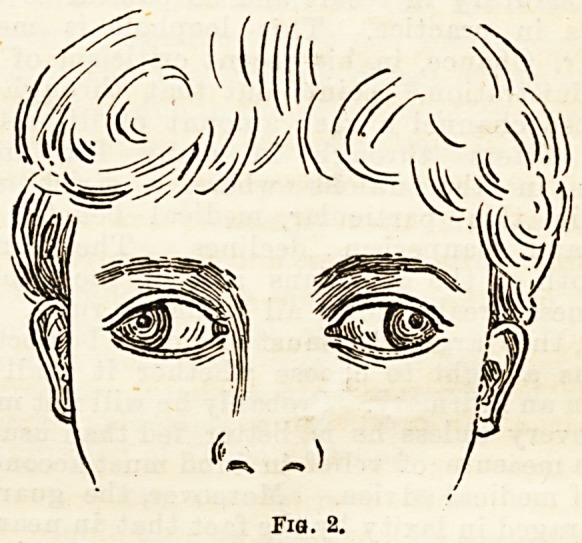# On Modern Progress in Ophthalmic Medicine and Surgery

**Published:** 1895-10-19

**Authors:** Robert Brudenell Carter

**Affiliations:** Consulting Ophthalmic Surgeon to St. George's Hospital.


### On Modern Progress in Ophthalmic Medicine and Surgery.

By Robert Brudenell Carter, F.R.C.S., Consulting Ophthalmic Surgeon to St. George's Hospital.

SQUINT (*Continued from page 3*).

As a further illustration of the unpleasant results which attended some of the early squint operations, I may mention that, many years ago, being on a visit to friends in a provincial town, I was asked to see a patient. Two ladies presented themselves at the appointed time, one small and loquacious, with her eyes in the position shown in Fig. 1, the other tall, silent, and thickly veiled. The second lady took a back seat, and held her peace, while the first assured me, as a fact, which, although hardly credible, was yet certain, that she was the subject of squint, and that she desired to be cured. Time and place having been fixed for the operation, both ladies rose, not, as I at first imagined, to depart, but only to change places. The second lifted her veil with much solemnity, revealing eyes in the position shown in Fig 2, and said, "I also squinted *once*, but seven doctors performed an operation upon me." I operated upon both ladies, and with satisfactory results; but the second of them afterwards expressed much regret that she had consented to my suggestion, because, she said, people looked at her so much as she went along the street. The fact was that her fellow-townsfolk wondered at her changed appearance, to which, no doubt, they would become reconciled in time.

Fig. 1.

Fig. 2.

Matters remained much where Dieffenbach and Bennett Lucas had left them until, about 1860, Professor Donders, of Utrecht, gradually arrived at and established the conclusion that squint, instead of being a primary affection of the ocular muscles, was merely a symptom, depending upon different causes in different cases, but usually upon some error of refraction—that is to say, upon some fault in the shape and optical conditions of the eyeball. The most common fault, and the cause of common convergent squint in perhaps nine-tenths of the cases of its occurrence, was found to reside in flatness of the eyeball, or, as Donders called it, hypermetropia. I prefer to use the term "flat eye."

The mechanism of the eye, as an optical instrument, is wholly analogous to that of a photographer's camera, in which a convex lens produces an image of the objects towards which it is directed, and casts this image upon a sensitive plate. Everyone knows that, if the lens and screen of the camera are in such a relation to each other that the screen receives a clear image of a distant object, this relation must be altered, and the lens moved farther away from the screen, in order to give an equally clear image of a near object. The same result might be attained, without any change of relative position, if the lens were rendered in some definite degree stronger. The former method is employed in the camera, the latter in the eye. The lens of the camera is set in a tube, which is made to slide in and out as required. The lens of the eye does not change its position in relation to its screen (the retina), but can be compressed and rendered stronger (or more convex) by a muscular apparatus with which the eye is provided. This apparatus is called the apparatus of accommodation, and the eye which uses it is said to accommodate.

In an eye which is of ideally correct proportions, and is usually called "normal," or "emmetropic," clear images of distinct objects are produced upon the retina when the accommodation is passive or at rest; and the accommodation is only called into activity in order to furnish equally clear images of near objects. In order to obtain single vision with the two eyes it is necessary that the clear images should be formed upon corresponding points of the retinæ, and for this purpose that the two eyes should be directed to the same point. For distant objects, the lines of direction are approximately parallel; but, as the objects of vision approach, these lines must of necessity become convergent. Hence, with normal eyes, the act of accommodation and the act of convergence are always required together. As the eyes turn to a near object, their accommodation is exerted; as they turn to a distant object, their accommodation is relaxed. The two movements, although depending on different stimuli, the accommodation on the requirement of distinct vision, the convergence on the requirement of single vision, have become, in the course of time and generations, so inseparably associated in the nerve centres that one can scarcely be performed without the other. Nor is this association liable to be disturbed, in any considerable degree, by a mere fault in the proportions of an external organ like the eye.

The flat eye, being too short from front to back, has its lens too near the screen which receives the image, so that this image is blurred and obscure, or "out of focus," when the eye is in a passive condition. In order to obtain a clear image, even of a distant object, the eye must exercise its accommodation, and that in a degree corresponding to, and sufficient to correct, the fault of shape of which its flatness is the expression. As the object approaches the eye a more and more strenuous effort of accommodation is required. This effort is attended by a corresponding convergence effort, by which the eyes are directed to a point nearer than that of the object looked at, and by the continued repetition of which the muscles effecting it become stronger than their antagonists, the external recti, and acquire a physiological preponderance which tends to keep the visual lines convergent, even when the eyes are at rest. In other words, the eyes become related in a position of convergence, instead of in the natural position of parallelism. The first effect would be to produce double vision of all objects beyond the point at which the visual lines would intersect each other if prolonged.

## Figures and Tables

**Fig. 1. f1:**
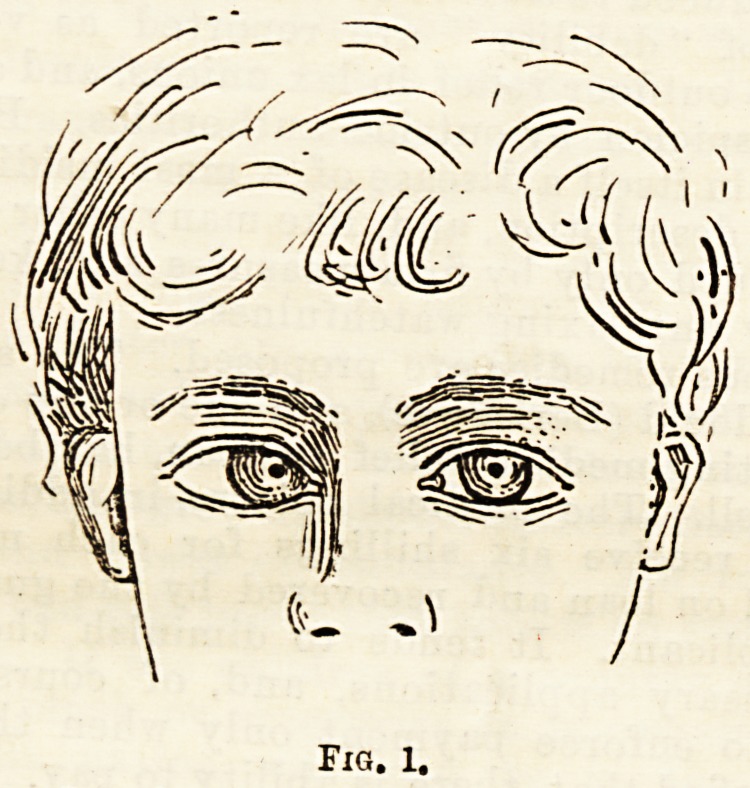


**Fig. 2. f2:**